# Supplementary figures and images for: Repetition velocity as a measure of loading intensity in the free weight and Smith machine Bulgarian split squat
Source: PeerJ. 2023 Aug 15;11:e15863. doi: 10.7717/peerj.15863 (PMC10437032; doi:10.7717/peerj.15863)

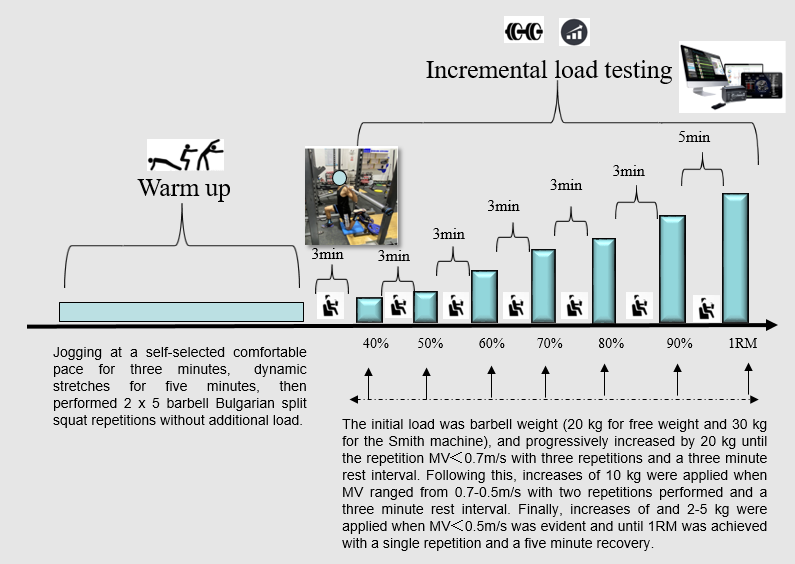

Supplement: Supplemental Information 2 [file peerj-11-15863-s002.png]
